# Food Image Recognition Based on Anti-Noise Learning and Covariance Feature Enhancement

**DOI:** 10.3390/foods14162776

**Published:** 2025-08-09

**Authors:** Zengzheng Chen, Hao Chen, Jianxin Wang, Yeru Wang

**Affiliations:** 1 School of Information, Beijing Forestry University, Beijing 100083, China; zengzheng@bjfu.edu.cn (Z.C.); chenhaoxdy@outlook.com (H.C.); 2 Risk Assessment Division 1, China National Center for Food Safety Risk Assessment, Beijing 100022, China

**Keywords:** food recognition, anti-noise learning, covariance feature enhancement, knowledge distillation, food science and technology

## Abstract

Food image recognition is a key research area in food computing, with applications in dietary assessment, menu analysis, and nutrition monitoring. However, imaging devices and environmental factors introduce noise, limiting classification performance. To address this, we propose a food image recognition method based on anti-noise learning and covariance feature enhancement. Specifically, we design a Noise Adaptive Recognition Module (NARM), which incorporates noisy images during training and treats denoising as an auxiliary task to enhance noise invariance and recognition accuracy. To mitigate the adverse effects of noise and strengthen the representation of small eigenvalues, we introduce Eigenvalue-Enhanced Global Covariance Pooling (EGCP) into NARM. Furthermore, we develop a Weighted Multi-Granularity Fusion (WMF) method to improve feature extraction. Combined with the Progressive Temperature-Aware Feature Distillation (PTAFD) strategy, our approach optimizes model efficiency without adding overhead to the backbone. Experimental results demonstrate that our model achieves state-of-the-art performance on the ETH Food-101 and Vireo Food-172 datasets. Specifically, it reaches a Top-1 accuracy of 92.57% on ETH Food-101, outperforming existing methods, and it also delivers strong results in Top-5 on ETH Food-101 and both Top-1 and Top-5 on Vireo Food-172. These findings confirmed the effectiveness and robustness of the proposed approach in real-world food image recognition.

## 1. Introduction

**Figure 1:**
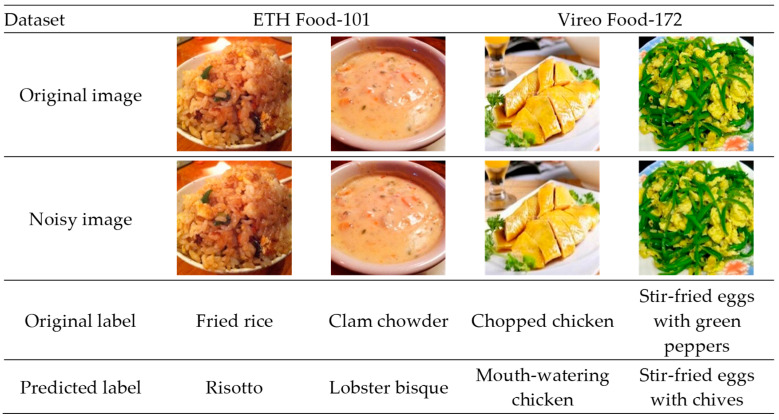
Examples of how noise causes recognition errors. The left column shows clear original images (e.g., fried rice and clam chowder), and the right column shows the same images with slight noise added (standard deviation of 0.01 Gaussian noise). To the human eye, the difference is almost unnoticeable, but standard models (like ResNet50 [16]) misclassify them (e.g., mistaking fried rice for risotto). These examples are from the ETH Food-101 [17] and Vireo Food-172 [18] datasets, highlighting why noise interference is a key problem our research aims to solve.

In daily life, food photos we take are often blurred by poor lighting or low-quality equipment, for example, stir-fries shot in a dim kitchen or salads captured with a shaky phone. These “noises” can cause recognition software to make mistakes, such as mistaking fried rice for risotto or clam chowder for lobster bisque (as shown in Figure 1). This creates significant troubles for practical applications. Food image recognition, a core technology in food computing, has broad prospects in diverse fields as follows: it supports dietary assessment, menu analysis, and nutrition tracking to help people monitor intake, analyze nutrients, and assist health management for chronic disease patients [1,2,3,4,5,6,7,8]; in the restaurant industry and food delivery platforms, it provides technical support for menu recommendations, dish classification, and food traceability, enhancing operational efficiency and user experience [9,10,11,12]; in the food industry, it aids in food classification, quality testing, and labeling, supporting intelligent modernization of production and processing workflows [13,14,15]. However, noise interference has long limited its practical use. Thus, enabling computers to “see food clearly amid noise” is the key problem this study aims to solve.

However, achieving high-accuracy food image recognition is not straightforward. This technology is a crucial subfield of fine-grained image recognition, which encounters the dual challenges of inter-class similarity and intra-class variability [19,20]. For instance, foods with similar appearances (e.g., salads and vegetable stir-fries) are prone to confusion, while differences within the same category due to different cooking methods (e.g., steamed fish vs. fried fish) further increase classification difficulty. To address these challenges, the “attention mechanism” is widely used by researchers to improve the effectiveness of food image recognition. The “attention mechanism” enhances classification accuracy by identifying and emphasizing important feature information in the image that is relevant to the classification task [21,22,23,24]. For example, some studies developed several attention modules that can be integrated into deep neural networks to improve focus on key features and enhance discriminative power [22,23,24]. Although the attention mechanism has successfully improved food image classification accuracy, its performance still faces some limitations. For instance, this mechanism is highly dependent on the accuracy of region localization, and its performance may deteriorate in complex backgrounds or noisy environments. Moreover, while many methods perform well in laboratory settings, they often lack the necessary robustness in practical applications, making it challenging to handle the diverse types of noise interference present in real-world scenarios.

Recent advances in food image recognition have focused on deep learning methods, including CNN-based architectures [25,26,27], attention mechanisms [21,22,23], and transformer-based models [28,29]. These approaches have achieved notable performance in benchmark datasets, yet they often rely on ideal image conditions and struggle under real-world challenges such as low lighting, motion blur, or sensor-induced noise. Moreover, fine-grained classification remains difficult due to the subtle visual differences among food categories and high intra-class variation.

In parallel, researchers have explored anti-noise techniques, ranging from classical filters like Block-Matching and 3D Filtering [30] to deep-learning-based restoration models such as Image Restoration Using Swin Transformer [31] and Denoising Diffusion Probabilistic Models [32]. While effective for image denoising, these methods are not optimized for classification tasks and may suppress discriminative features critical for recognition.

These limitations highlight the need for an integrated approach that jointly addresses noise robustness and fine-grained classification. To this end, we propose a novel food image recognition framework that combines noise-adaptive learning, covariance feature enhancement, and multi-granularity feature fusion. Our contributions include the design of a Noise Adaptive Recognition Module (NARM), the use of Eigenvalue-Enhanced Global Covariance Pooling (EGCP), and a lightweight deployment strategy via Progressive Temperature-Aware Feature Distillation (PTAFD). This approach aims to improve recognition accuracy and robustness under noisy, real-world conditions, laying the foundation for practical dietary assessment applications.

## 2. Materials and Methods

### 2.1. Datasets

To validate the effectiveness of the proposed method, we evaluated our model on two popular food datasets, ETH Food 101 [17] and Vireo Food-172 [18].

ETH Food-101 is a classic dataset containing 101 categories of Western foods with 101,000 images. Each category includes 1000 images, divided into 750 training and 250 test images at a 3:1 ratio. The images in this dataset come from different angles, lighting conditions, and backgrounds, offering high diversity and challenges. ETH Food-101 is unprocessed; researchers can crop, scale, and normalize the images. As a widely used benchmark for image classification, feature extraction, and transfer learning, it provides a reliable evaluation basis for fine-grained food image classification tasks.

Vireo Food-172 is a dataset focused on Chinese cuisine, comprising 172 classes of Asian dishes with a total of 110,241 images. The dataset is split into training, validation, and test sets at a ratio of 60%, 10%, and 30%, respectively. The design of Vireo Food-172 aims to advance the development of food image classification tasks in computer vision. The number of images per category in this dataset is imbalanced, reflecting the data distribution in real-world applications. It offers a wealth of varied visual data, featuring multiple camera angles and backgrounds. This makes it highly suitable for both training and evaluating deep learning models designed for food recognition tasks.

### 2.2. Modification of the Original Dataset

In the experiment, ResNet50 [16] was used as the backbone network, and it consists of five stages. In its last three stages, we added three NARMs, so in Algorithm 1, the parameter S is set to 3. To highlight the importance of deep layers, the feature fusion weights $\beta_{i}$ of these three network layers are set to 0.2, 0.35, and 0.45, respectively. In the PTAFD framework, we also use these three intermediate feature maps to transfer knowledge, with the weighting coefficient α for the student network’s classification loss set to 0.5.

This study assessed the model’s classification accuracy and its resilience to noise using the ETH Food 101 and Vireo Food-172 datasets. Data preparation adhered to the following three steps: first, resize images to 550 × 550; then, center-crop them to 448 × 448; and finally, normalize the pixels. For training, the model runs for 200 epochs with a batch size of 32. We used stochastic gradient descent (SGD) as the optimizer, starting with a learning rate of 0.002 and adjusting it via a cosine annealing schedule. The hyperparameters D, $D^{\prime }$, and $D^{\prime \prime }$ are used to control the number of channels in the convolutional and fully connected layers, set to 1024, 256, and 64, respectively. To evaluate noise resilience, Gaussian noise with a mean of 0 and standard deviation of 0.10 (determined optimal through ablation experiments in Section 3.2) is added to the images, simulating real-world image degradation caused by sensor noise or low-light conditions.

The evaluation metrics adopted in this study include Top-1 and Top-5 classification accuracy. All neural network models are implemented using the PyTorch framework. The programming environment is based on Python 3.10, with the primary libraries being torchvision 0.15.2 + cu118 and torch 2.0.1 + cu118. Experimental training is conducted on a hardware setup comprising eight NVIDIA GeForce RTX 3090 GPUs (NVIDIA Corporation, Santa Clara, CA, USA), each equipped with 24 GB of memory and operated in parallel.

### 2.3. Neural Network Architecture

This research introduces several innovative approaches to tackle the noise problems in food image classification and restoration, including the Noise Adaptive Recognition Module (NARM), the Weighted Multi-Granularity Fusion (WMF) method, and the Progressive Temperature-Aware Feature Distillation (PTAFD) method. These methods are designed to address noise processing, feature fusion, and model efficiency optimization, with detailed explanations provided in the following sections.

#### 2.3.1. Noise Adaptive Recognition Module (NARM)

**Figure 2:**
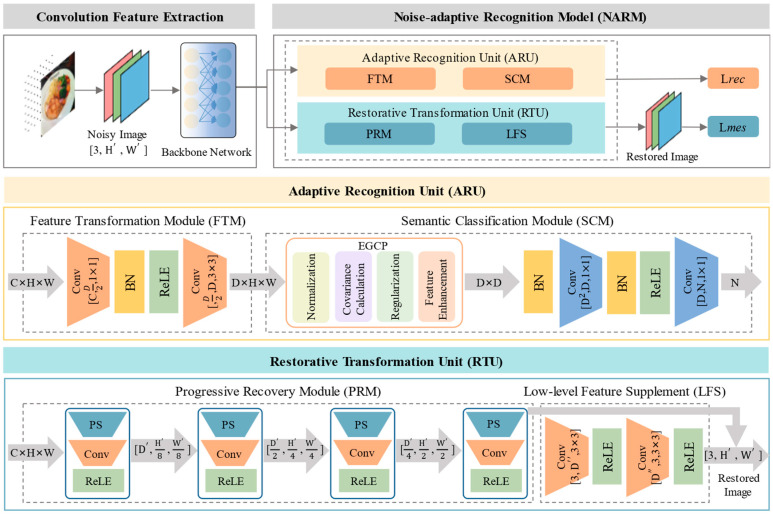
The architecture of the NARM is shown, which includes the Adaptive Recognition Unit (ARU) and the Recovery Transformation Unit (RTU). The Adaptive Recognition Unit (ARU) consists of the Feature Transformation Module (FTM) and the Semantic Classification Module (SCM). The Recovery Transformation Unit (RTU) includes the Progressive Recovery Module (PRM) and the Low-level Feature Supplement (LFS) module. D, $D^{\prime }$, and $D^{\prime \prime }$ serve as hyperparameters that regulate the number of channels within both the convolutional and fully connected layers. $H^{\prime }\times W^{\prime }$ is the image size input into the backbone network.

Figure 2 shows the design of the NARM. Initially, Gaussian noise was injected into the original image to generate a noisy image. The noisy image is subsequently fed into the CNN backbone network. In this paper, the backbone network uses the ResNet50 architecture. The output feature vector from the backbone network is input into both the ARU and the RTU for image recognition and denoising.

Adaptive Recognition Unit (ARU): ARU consists of the Feature Transformation Module (FTM) and the Semantic Classification Module (SCM). For the feature vector $x\in {\mathbb{R}}^{C\times H\times W}$ from the backbone network, it is first input into the FTM for preliminary feature extraction. Then the extracted results are passed to the SCM for covariance feature enhancement and final classification. The FTM consists of multiple convolutional layers, followed by Batch Normalization and Rectified Linear Unit (ReLU) activation functions. Specifically, the formula is given as follows:

(1) $$d_{1}={\mathrm{Conv}}_{1\times 1}(x),\;d_{1}\in {\mathbb{R}}^{\frac{D}{2}\times H\times W},$$

(2) $$d_{1}^{\prime }=\mathrm{ReLU}(\mathrm{BN}(d_{1})),\;d_{1}^{\prime }\in {\mathbb{R}}^{\frac{D}{2}\times H\times W},$$

(3) $$d_{2}={\mathrm{Conv}}_{3\times 3}(d_{1}^{\prime }),\;d_{2}\in {\mathbb{R}}^{D\times H\times W}.$$

In Equation (1), the size of the convolutional kernel filter is denoted as $\left[C,\frac{D}{2},1\times 1\right]$. In Equation (3), the size of the convolutional kernel filter is denoted as $\left[\frac{D}{2},D,3\times 3\right]$. The resulting preliminary feature extraction output is denoted as $d_{2}\in {\mathbb{R}}^{D\times H\times W}$.

In the SCM, for $d_{2}\in {\mathbb{R}}^{D\times H\times W}$, the feature value enhancement is first performed using EGCP, and then the final classification task is completed through a series of convolutions, batch normalization, and ReLU operations. The EGCP process consists of the following four steps: feature normalization, weighted covariance matrix computation, covariance matrix regularization, and feature enhancement mechanism. Specifically, the introduction of feature normalization helps balance the feature distribution, thereby effectively reducing the impact of noise on covariance matrix computation as follows:

(4) $$d_{2,d}^{\mathrm{norm}}(h,w)=\frac{d_{2}(d,h,w)-\mu_{d}}{\sqrt{\sigma_{d}^{2}+\epsilon }},$$

where $\mu_{d}$ represents the mean of the d-th channel, $\sigma_{d}$ represents the standard deviation of the d-th channel, and ϵ is a positive number to prevent division by zero. Through the above process, the normalized feature vector $d_{2,d}^{\mathrm{norm}}$ is obtained. Subsequently, we compute the weighted covariance matrix to adjust the importance of feature channels dynamically, as follows:

(5) $$w_{d}=\frac{1}{\sigma_{d}^{2}+\epsilon },$$

(6) $$P=\sum_{d=1}^{D}w_{d}\left(d_{2,d}^{\mathrm{norm}}-\mu \right){\left(d_{2,d}^{\mathrm{norm}}-\mu \right)}^{T},$$

where $w_{d}$ represents the weight, and the larger the variance of a channel, the smaller its corresponding weight, effectively reducing the noise impact that high-variance channels may introduce. The resulting $P\in {\mathbb{R}}^{D\times D}$ expresses the second-order correlation between feature channels. To improve the numerical stability of the covariance matrix and avoid the occurrence of ill-conditioned matrices, we regularize P, and perform eigenvalue decomposition on the regularized covariance matrix, as follows:

(7) $$P_{\mathrm{reg}}=P+\epsilon I,$$

(8) $$P_{\mathrm{reg}}=U\Lambda U^{T},$$

where I is the identity matrix, and U is the eigenvector matrix, which results in the feature matrix $\Lambda =\mathrm{diag}(\lambda_{1},\lambda_{2},\ldots ,\lambda_{D})$. To avoid numerical instability, we perform a lower bound correction on the eigenvalues to ensure the stability of the computation as follows:

(9) $$\lambda_{i}^{\mathrm{reg}}=\max(\lambda_{i},\epsilon ).$$

The core mechanism of the EGCP module is to enhance the small eigenvalues in the covariance matrix, thereby improving feature discriminability in classification tasks. Specifically, the EGCP module first uses a logarithmic function for the nonlinear transformation of the eigenvalues, adjusting the eigenvalues of the regularized covariance matrix as follows:

(10) $$\lambda_{i}^{\mathrm{en}}=\log(1+\lambda_{i}).$$

Then, the enhanced eigenvalues are used to construct the improved covariance matrix as follows:

(11) $$P_{\mathrm{en}}=U\Lambda_{\mathrm{en}}U^{T}.$$

This results in the enhanced diagonal eigenvalue matrix $\Lambda_{\mathrm{en}}$. However, in practical applications of food images, due to the significant differences in feature distributions across different foods, relying solely on the enhancement mechanism may lead to uneven feature distributions, which in turn affects classification performance. To alleviate this issue, we design a dynamic scaling factor mechanism for the EGCP module, which adaptively adjusts the enhancement strength to make the enhanced feature distribution more uniform and stable, thereby improving classification performance. First, construct the dynamic scaling matrix as follows:

(12) $$S=\exp(-P_{\mathrm{en}})=U\exp(-\Lambda_{\mathrm{en}})U^{T}.$$

The differences between eigenvalues are effectively balanced by taking the inverse exponential matrix of the enhanced covariance matrix. Then, the cross-covariance matrix is computed, and the dynamic scaling factor is defined based on the Frobenius norm of the matrix as follows:

(13) $$Q_{\mathrm{cross}}=P_{\mathrm{en}}^{1/2}\cdot S,$$

(14) $$\mathrm{SF}=\|Q_{\mathrm{cross}}{\|}_{F}=\sqrt{\sum_{i=1}^{D}{\left(\lambda_{i}^{1/2}e^{-\lambda_{i}}\right)}^{2}},$$

where D is the feature dimension, and $\lambda_{i}$ is the enhanced eigenvalue. By introducing the dynamic scaling factor, the EGCP module can further adjust the feature distribution to accommodate the complex feature distributions of different food types. Ultimately, the EGCP module and the dynamic scaling factor further optimize the enhanced covariance matrix, producing the final feature representation. Specifically, the final feature matrix is represented as follows:

(15) $$A=(\mathrm{SF}+1)\cdot P_{\mathrm{en}}^{1/2},$$

and the dimension of the feature matrix A is D×D,which can be flattened into ${\mathbb{R}}^{D^{2}}$. In the final stage of the ARU, we complete the final classification task through a series of convolution operations, Batch Normalization, and ReLU activation functions, as expressed by the following formulas:

(16) $$f_{1}^{\prime }=\mathrm{ReLU}(\mathrm{BN}({\mathrm{Conv}}_{1\times 1}(A))),\;f_{1}^{\prime }\in {\mathbb{R}}^{D\times H\times W},$$

(17) $$f_{2}={\mathrm{Conv}}_{1\times 1}(d_{1}^{\prime }),\;f_{2}\in {\mathbb{R}}^{D\times H\times W},$$

where, in Equation (16), the convolution kernel filter size is $\left[D^{2},D,1\times 1\right]$. In contrast, in Equation (17), the convolution kernel filter size is [D,N,3×3]. Here, $p\in {\mathbb{R}}^{N}$ denotes the predicted score, where N denotes the overall number of food classifications.

Restorative Transformation Unit (RTU): RTU consists of the Progressive Recovery Module (PRM) and the Low-level Feature Supplement (LFS) module. The former is responsible for extracting image information from the intermediate features of the backbone network. At the same time, the latter is used to supplement detailed information from noisy inputs, achieving comprehensive recovery from noisy images to clear images. PRM mainly recovers the clean image through progressive upsampling operations from the feature vector output by the backbone network. PRM is composed of multiple progressively connected upsampling modules, and the definition of each module is given by the following:

(18) $$M_{k}^{\mathrm{up}}(x)=\mathrm{ReLU}(\mathrm{Conv}(\mathrm{PixelShuffle}(x))).$$

PRM is composed of four such modules, and the specific output is given by the following:

(19) $$I_{\mathrm{PRM}}=\mathrm{PRM}(x)=M_{4}^{\mathrm{up}}(M_{3}^{\mathrm{up}}(M_{2}^{\mathrm{up}}(M_{1}^{\mathrm{up}}(x)))).$$

Through the progressive recovery process, PRM ultimately changes the resolution from H×W to the target size $H^{\prime }\times W^{\prime }$, and the output of the recovered image is $I_{PRM}\in {\mathbb{R}}^{3\times H^{\prime }\times W^{\prime }}$.

Since intermediate features of the backbone network may lose low-level detail information during the extraction process, LFS directly extracts low-level features from the input noisy image to supplement this crucial information. LFS is implemented through two shallow convolution operations, and the specific formula is as follows:

(20) $$I_{\mathrm{LFS}}=\mathrm{LFS}(I_{\mathrm{noisy}})={\mathrm{Conv}}_{2}({\mathrm{Conv}}_{1}(I_{\mathrm{noisy}})),$$

where $I_{\mathrm{noisy}}\in {\mathbb{R}}^{3\times H^{\prime }\times W^{\prime }}$ is the input noisy image. The outputs of PRM and LFS were fused by pixel-wise addition to generate the final recovered image as follows:

(21) $$I_{\mathrm{restored}}=I_{\mathrm{PRM}}+I_{\mathrm{LFS}},$$

where $I_{\mathrm{restored}}$ is the final clean image generated, combining the global details recovered by PRM with the low-level features supplemented by LFS.

The initial component involves the Softmax loss calculated between the classification output $p\in {\mathbb{R}}^{N}$ and the target label. The second part is the pixel-wise Mean Square Error (MSE) between the recovered image $I_{\mathrm{restored}}$ and the clean image $I_{clear}$ without noise injection. The calculation method is as follows:

(22) $$L_{\mathrm{rec}}=\mathrm{Softmax}(p,g),$$

(23) $$L_{mse}=\mathrm{MSE}(I_{\mathrm{restored}},I_{\mathrm{clean}}),$$

(24) $$L_{NARM}=\alpha L_{rec}+\beta L_{mse},$$

where α and β are balancing parameters, producing the final loss function $L_{NARM}$.

#### 2.3.2. Weighted Multi-Granularity Fusion (WMF)

To significantly improve the representational capacity of features across various stages of the backbone network, we propose Weighted Multi-Granularity Fusion (WMF), which improves recognition task performance by integrating features from different depth layers.

In the backbone network, layers at varying depths capture information at different levels of abstraction. Early layers in the network tend to capture fine-grained and specific features, whereas deeper layers emphasize more abstract and semantically rich information. For recognition tasks, the training process is typically dominated by deep-layer features. In comparison, for low-level vision tasks like image restoration, shallow-layer features generally have a more substantial impact unless an advanced mechanism is used to balance deep and shallow features.

This paper emphasized recognition as the primary objective while incorporating image restoration as a secondary supportive task. This approach ensures that image restoration does not overly focus on superficial features. To address this, we adopted a stepwise training approach, first training the shallow layers, then gradually progressing to deeper layers, and ultimately completing the training of the deepest layer. During the training process, by minimizing each loss gradually rather than optimizing them simultaneously, the model can more effectively learn the features of each layer.

After training each NARM separately, we need to integrate their outputs. This is where WMF comes in; it combines features from all stages with different weights. By doing so, the model synthesizes both low-level details (like color) and high-level semantics (like category), avoiding over-reliance on single-layer features and thus improving overall recognition performance.

Specifically, as shown in Algorithm 1, we insert S NARMs into the backbone network, dividing the entire training process into S + 1 steps. In the first S steps, these S NARMs receive feature vectors from the backbone network and process them to optimize the loss functions $L_{rec}$ and $L_{mse}$. This phase concentrates on extracting and optimizing features via each NARM, allowing the model to efficiently capture and represent essential information and characteristics from the input data.

In step S + 1, we use the weighted fusion method to integrate the feature maps from all previous stages. First, the feature maps from all stages are weighted and fused to generate a multi-granularity fused feature map, as follows:

(25) $$x^{*}=\sum_{i=1}^{s}\beta_{i}x_{i},$$

where $\beta_{i}$ is a hyperparameter used to adjust the contribution of each stage’s feature map to the final fused feature map. By appropriately setting this hyperparameter, we can flexibly control the significance of feature maps from different stages. Finally, for the resulting fused feature map $x^{*}$, we used the ARU in EGCP to compute the loss function.

**Algorithm 1** Weighted Multi-Granularity Fusion**Require**: Given a dataset $D{=\text{\{}(\mathrm{input}}^{i}{,\mathrm{target}}^{i}{)\text{\}}}_{i=1}^{I}$ (where I represents the total number of batches in D)1:**for** epoch = 1 to num_of_epochs **do**2: **for** (input, target) in D **do**3:  **for** n = 1 to S **do**4:   $x_{i}=\{x_{1},x_{2},\ldots ,x_{s}\}$5:   # NARM6:   $L_{\mathrm{NARM}}^{\left(i\right)}=\alpha L_{\mathrm{rec}}^{\left(i\right)}+\beta L_{\mathrm{mse}}^{\left(i\right)}$7:   **BACKWARD**($L_{\mathrm{NARM}}^{\left(i\right)}$)8:  **end** **for**9:  # WMF10:  $x^{*}=\sum_{i=1}^{s}\beta_{i}x_{i}$11:  $L_{\mathrm{cls}}=\mathrm{ARU}(x^{*})$12:  **BACKWARD**($L_{\mathrm{cls}}$)13: **end** **for**14:**end** **for**

#### 2.3.3. Progressive Temperature-Aware Feature Distillation (PTAFD)

As discussed earlier, multiple NARMs are introduced in WMF to enhance the model’s feature extraction capability, and a multi-step training strategy is adopted. However, this method inevitably adds extra computational costs. At the same time, with the introduction of new modules, the computational complexity in the backpropagation process also increases significantly, which impacts the overall training efficiency. In practical applications, it is crucial to recognize that the efficiency during the inference stage frequently holds greater importance compared to the training stage. This necessitates a careful balance between the complexity involved in training and the efficiency achieved during inference.

To tackle this challenge, we introduce a Progressive Temperature-Aware Feature Distillation (PTAFD) approach that integrates temperature regulation with a step-by-step learning framework for knowledge distillation. The core concept of PTAFD is to introduce a temperature control mechanism into the knowledge distillation process and use a progressive learning strategy to gradually optimize the student model’s absorption of knowledge from the teacher model via a hybrid loss function that combines soft predictions from the teacher with the true labels, guided by a progressive temperature adjustment strategy.

**Figure 3:**
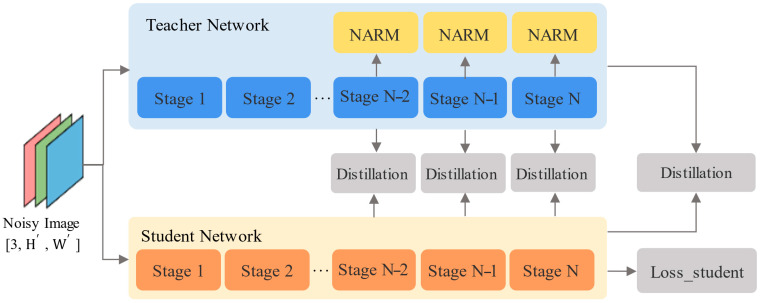
Schematic diagram of Progressive Temperature-Aware Feature Distillation (PTAFD). The teacher network contains multiple NARMs, with each network layer corresponding to the respective layer in the student network.

We adjust the degree of attention the student model pays to labels in a staged manner, gradually improving its learning efficiency. As illustrated in Figure 3, a network comprising multiple NARMs and WAF serves as the teacher model, while a conventional CNN backbone network acts as the student model. It is crucial to observe that both the teacher and student models employ the same backbone network structure, ensuring they share an equal number of stages.

Specifically, during training, the teacher model generates soft labels using the Softmax function as follows:

(26) $${\hat{y}}_{\mathrm{teacher}}=\mathrm{softmax}\left(\frac{x_{\mathrm{teacher}}}{T}\right),$$

where $x_{\mathrm{teacher}}$ is the final feature used by the teacher model for classification, and T is the temperature parameter. When T<1, the output distribution becomes sharper; when T>1, the output distribution becomes smoother. During training, the student model is divided into the following two branches: the soft prediction branch and the complex prediction branch, which are used to calculate the distillation loss and the classification loss of the student network, as follows:

(27) $$L_{\mathrm{distillation}}=-\sum_{i}{\hat{y}}_{\mathrm{teacher},i}\log({\hat{y}}_{\mathrm{student},i}),$$

(28) $$L_{\mathrm{student}}=-\sum_{j}y_{j}\log({\hat{y}}_{\mathrm{student},j}),$$

where ${\hat{y}}_{\mathrm{student}}$ represents the soft labels of the student model, y represents the actual labels, and $L_{\mathrm{student}}$ is the output of the student model when T=1. The variable i is used to identify the current category being evaluated, while j is used to determine the index of the true class in the complex labels. By combining the two losses above, weighted by coefficient α, the total loss function is formed as follows:

(29) $$L=\alpha \cdot L_{\mathrm{distillation}}+(1-\alpha )\cdot L_{\mathrm{student}}.$$

The choice of temperature and the implementation of a progressive strategy are essential throughout various phases of the training process. PTAFD adjusts the temperature parameter in stages to optimize learning. In the initial stage, it uses a lower temperature T (T<1). This makes the student model focus on high-probability labels from the teacher, reducing interference from incorrect labels. Later, the temperature is gradually increased (from 1 to 2), which provides a controlled smoothing effect on the teacher model’s soft labels. This range was chosen to preserve discriminative confidence while exposing inter-class relationships without over-smoothing the output distribution. This shift encourages the student to pay more attention to negative labels, enabling more comprehensive knowledge absorption. By dynamically adjusting the temperature and weighting coefficient, PTAFD can improve the student model’s performance in complex classification tasks and effectively utilize information from negative labels.

In the PTAFD approach, the training procedure is split into two phases. During the initial phase, referred to as the distillation phase, the primary objective is to progressively refine the feature representation of the student network by leveraging feature distillation techniques. In this stage, we extract features from each corresponding stage of the teacher and student networks. We use a distillation loss function to align their feature distributions for efficient knowledge transfer from the teacher network. Specifically, we introduce a temperature-aware mechanism, making the distillation process progressive, as follows: in the early stages, high temperature is used to align coarse-grained feature representations, helping the student network capture global features; in the later stages, the temperature is reduced to focus on fine-grained feature alignment, encouraging the student network to more precisely learn high-quality feature representations. Once the distillation phase is completed, the student model no longer relies on the teacher model’s guidance. At this stage, the student model is trained using a standard cross-entropy loss between its predicted outputs and the ground-truth labels, following the distillation phase.

### 2.4. Experimental Design

To comprehensively evaluate the proposed food recognition method, we designed four groups of experiments as follows: comparison with state-of-the-art methods, ablation studies of key modules, assessment of noise intensity, and evaluation of distillation strategies.

All experiments were conducted on the ETH Food-101 and Vireo Food-172 datasets, using the same preprocessing pipeline and data partitioning described in Section 2.1 (i.e., ETH: 75% training/25% test; Vireo: 60% training/10% validation/30% test). Each image was resized to 550 × 550, center-cropped to 448 × 448, and normalized using ImageNet mean and standard deviation.

Unless otherwise specified, all experiments shared the following identical training configuration: batch size of 32, 200 training epochs, stochastic gradient descent (SGD) optimizer with an initial learning rate of 0.002, and a cosine annealing learning rate schedule. This unified setup ensures consistency and comparability across experimental conditions.

#### 2.4.1. Comparison with State-of-the-Art Methods

This experiment aimed to benchmark our method against representative food recognition models, including both CNN-based (e.g., ResNet50 [16], DenseNet161 [33], PRENet [34]) and transformer-based (e.g., SICL [29], IVRDRM [35]) architectures.

The independent variable was the recognition method (i.e., model architecture and key components), while the dependent variables were Top-1 and Top-5 classification accuracy. Evaluations were conducted on the test sets of ETH Food-101 (25,250 images) and Vireo Food-172 (33,072 images) under identical training conditions, ensuring fair comparison. All models were trained from scratch using the same data splits and preprocessing settings described above.

#### 2.4.2. Ablation Study Design

To evaluate the contributions of the proposed modules and strategies, we conducted six ablation experiments. First, to assess the individual and combined effects of EGCP and WMF, we compared the following four variants of our model: the baseline SFF (using only final-stage features), SFF with NARM (without EGCP), SFF with full NARM (including EGCP), and the complete model integrating both NARM and WMF. This experiment aimed to determine whether the covariance enhancement and multi-granularity fusion mechanisms lead to better recognition performance.

Next, we evaluated the role of RTU in enhancing noise robustness. For this, we constructed two models—one including both ARU and RTU and another with ARU alone—and tested their classification accuracy under increasing levels of Gaussian noise added to the test images. This helped verify whether the recovery path provided by RTU effectively mitigates the impact of noise during inference.

We also examined where to best insert the NARMs within the ResNet50 backbone. Five model variants were tested, each inserting NARMs at a different stage (from stage 1 to stage 5). This allowed us to explore how semantic depth affects the balance between noise-invariant learning and computational efficiency.

To understand the computational trade-offs introduced by each module, we compared parameter size, memory usage, and training time across the following four model configurations: SFF, SFF + NARM, SFF + NARM + WMF, and the PTAFD student model. This evaluation provides practical insights into the efficiency–performance balance for potential deployment scenarios.

Additionally, we studied how different loss weightings affect the model’s learning behavior. By varying the balance between classification and reconstruction loss (e.g., α:β = 1:0, 0.6:0.4, 0:1), we assessed how the network prioritizes high-level semantics versus low-level recovery and its impact on final recognition accuracy.

Finally, we analyzed per-class performance for the full model and its ablated variants to better understand their behavior across diverse food types. We focused on the first ten classes in the ETH Food-101 dataset and reported per-class F1-scores and recall to observe how EGCP and WMF affect the recognition of visually similar and minority classes.

#### 2.4.3. Effect of Noise Intensity

To further examine the model’s robustness to varying levels of image degradation, we trained the network under different levels of injected additive Gaussian noise, with standard deviation (σ) values ranging from 0.01 to 0.20. The test sets remained clean across all conditions to simulate practical deployment scenarios. This setup allowed us to identify whether moderate noise serves as a form of regularization and how performance deteriorates when noise becomes excessive. All other training configurations were kept constant to ensure fair comparison.

#### 2.4.4. Evaluation of Distillation Strategies

We compared our proposed Progressive Temperature-Aware Feature Distillation (PTAFD) method with several mainstream knowledge distillation approaches, including feature alignment methods and distribution-based methods. All experiments used the same teacher model—our full architecture with NARM and WMF—and the same ResNet50-based student network. The goal was to determine whether PTAFD offers more effective knowledge transfer under complex food classification tasks, particularly in the presence of noise and fine-grained visual categories. Each method was trained and evaluated five times to ensure reliability.

### 2.5. Performance Metrics

To evaluate the effectiveness and robustness of our method, we employed several standard classification metrics during testing, including Top-1 accuracy, Top-5 accuracy, recall, and F1-score [36,37]. These metrics were selected to provide both overall and per-class performance insights, especially in imbalanced or fine-grained recognition settings.

Top-1 accuracy is defined as the proportion of test samples for which the top predicted label matches the ground truth. Formally, given N test samples, the Top-1 accuracy is computed as follows:

(30) $$\mathrm{Top}\text{-}1\;\mathrm{Acc}=\frac{1}{N}\sum_{i=1}^{N}I(y_{i}={\hat{y}}_{i}),$$

where $y_{i}$ is the ground truth label, ${\hat{y}}_{i}$ is the top-1 predicted label, and I(⋅) is the indicator function.

Top-5 accuracy measures the proportion of test samples where the correct label appears among the model’s top five predictions. It is defined as follows:

(31) $$\mathrm{Top}\text{-}5\;\mathrm{Acc}=\frac{1}{N}\sum_{i=1}^{N}I(y_{i}\in {\hat{Y}}_{i}^{\left(5\right)})$$

where ${\hat{Y}}_{i}^{\left(5\right)}$ is the set of top-5 predicted labels for sample i.

In the Per-Class Performance Analysis of Ablation Modules, we further report the recall and F1-score to provide fine-grained insights into model behavior across different categories, especially for minority or visually similar classes. Recall is defined as the proportion of correctly predicted positive instances over the total number of actual positive instances for a given class c, as follows:

(32) $${\mathrm{Recall}}_{c}=\frac{TP_{c}}{TP_{c}+FN_{c}},$$

where $TP_{c}$ and $FN_{c}$ denote the number of true positives and false negatives for class c, respectively. The F1-score is the harmonic mean of precision and recall, and it is calculated per class as follows:

(33) $${F1}_{c}=\frac{2\cdot {\mathrm{Precision}}_{c}\cdot {\mathrm{Recall}}_{c}}{{\mathrm{Precision}}_{c}+{\mathrm{Recall}}_{c}},$$

(34) $${\mathrm{Precision}}_{c}=\frac{TP_{c}}{TP_{c}+FP_{c}},$$

where $FP_{c}$ is the number of false positives for class c.

In this study, we report the macro-averaged F1-score, which treats all classes equally by averaging the F1-scores over all classes. To ensure statistical reliability, all experiments were independently repeated five times, using different random seeds. The final results are reported as the mean ± standard deviation, reflecting the performance variability across runs and supporting the reproducibility of the proposed method.

## 3. Results

### 3.1. Comparison with Baselines

To verify the effectiveness of our method in food category recognition tasks, we compared our model with state-of-the-art food recognition methods on ETH Food-101 and Vireo Food-172. We also compare the backbone networks of different techniques for a fair comparison. Table 1 presents the performance outcomes on the ETH Food-101 and Vireo Food-172 datasets. For the ETH Food-101 dataset, our approach achieved notable results in both Top-1 and Top-5 classification accuracy under identical experimental settings. It was evident that our model surpasses the ResNet50 backbone network by 5.15% in Top-1 accuracy and by 1.30% in Top-5 accuracy. Additionally, compared to PRENet, which employs the same backbone architecture, our method demonstrates improvements of 2.66% in Top-1 accuracy and 0.66% in Top-5 accuracy.

On the ETH Food-101 dataset, our method performs best in Top-1 accuracy, while in Top-5 accuracy, it is second only to SICL with CBiAFormer-B as the backbone, primarily due to the advantages of CBiAFormer-B’s network architecture. The network uses the more advanced Swin-B architecture, providing more substantial feature extraction and modeling capabilities. Additionally, CBiAFormer-B integrates a dual-branch adaptive attention mechanism and a category-component cross-task interaction module (SICL), which can fully model the complex interaction between components and categories, thus effectively capturing diverse features and subtle semantic relationships in images, achieving excellent performance in Top-5 recognition tasks. However, CBiAFormer-B does not adopt lightweight techniques, so its model complexity is relatively high, which may make it less convenient for deployment in resource-constrained environments. In contrast, our method introduces distillation techniques, effectively reducing model complexity while maintaining good performance, demonstrating the advantages of our approach in flexibility and adaptability.

We can also observe that although some fine-grained methods perform better than the baseline network, their results on food datasets are not as strong as on standard fine-grained datasets. For example, DCL performs worse on ETH Food-101 compared to other fine-grained datasets, possibly because it fails to fully consider texture information from shallow networks and the differences in feature distributions within the same category. Furthermore, certain fine-grained approaches, like PMG, exhibit inferior performance compared to the baseline network. This is attributed to PMG’s inadequate emphasis on local feature interactions and its limited capability to facilitate effective multi-level feature learning. This causes the model to focus on standard semantic features. However, the non-rigid structure of many food categories and the lack of fixed semantic information make it difficult for these methods to perform well. The experimental findings indicate that simply utilizing existing fine-grained approaches does not ensure optimal performance in food recognition. This highlights the adaptability and effectiveness of our proposed model for fine-grained food recognition.

On the Vireo Food-172 dataset, our approach achieves higher Top-1 and Top-5 classification accuracy compared to the majority of food recognition methods, placing second only to IVRDRM. Despite IVRDRM utilizing the stronger ResNet-101 backbone and achieving outstanding performance with Top-1 accuracy of 93.33% and Top-5 accuracy of 99.15% through multi-task learning and component-region discovery combined with graph relationship modeling, our method also performs well with the ResNet-50 backbone, reaching 92.37% Top-1 accuracy and 98.55% Top-5 accuracy. Despite using a less complex backbone, this indicates that our model significantly improves classification robustness and accuracy in complex scenarios by combining anti-noise learning modules and multi-granularity feature fusion. The higher accuracy of IVRDRM is mainly due to its strong backbone network and deep modeling of component relationships, whereas our model emphasizes improving stability and robustness through anti-noise learning and feature enhancement modules. These results demonstrated that our method effectively controls model complexity while improving classification performance.

Furthermore, the Vireo Food-172 dataset suffers from class imbalance. When there are fewer samples in specific categories, the model struggles to learn sufficient features, resulting in poor recognition performance for those categories. This data imbalance significantly affects the model’s accuracy on minority classes, thereby reducing overall classification performance. Nonetheless, our method demonstrates strong robustness in addressing the data imbalance problem, and its overall recognition performance surpasses most existing methods.

### 3.2. Ablation Study Results

In this section, we perform a comprehensive ablation study to assess the contribution of key modules and design choices to the model’s overall performance. We begin by analyzing the individual effects of NARM and WMF, followed by evaluating the benefit of RTU for noise invariance. Next, we examine how different insertion stages of NARM within the backbone network affect recognition accuracy. We then assess the computational efficiency of the proposed components and investigate how varying the loss weight configuration impacts performance. Finally, a per-class performance analysis highlights the role of each module in handling visually similar and minority food categories. These studies provide a systematic understanding of how each component contributes to robustness, accuracy, and efficiency.

NARM and WMF Contribution Analysis Experiment: In this module contribution analysis experiment, we aimed to investigate NARM and WMF’s contribution to the model’s overall performance. To validate the effectiveness of the EGCP module in NARM, we designed an experiment where the model uses the NARM architecture but excludes the EGCP module. We also compared the basic ResNet50 model, which only uses the last three feature layers, with other settings consistent with the final model design, called “Simple Feature Fusion” (SFF). The experimental results are shown in Table 2, and we can observe the following: (1) Compared to SFF, our final model shows a notable improvement in Top-1 classification accuracy on both datasets. (2) After introducing the NARM on top of SFF, the model with the EGCP module achieved Top-1 classification accuracy improvements of 1.88% and 2.16% on the two datasets, compared to the network without the EGCP module. This indicates that EGCP, by providing global feature covariance, effectively enhances the model’s feature extraction and noise robustness, thereby improving classification performance. (3) With the introduction of the EGCP module, the classification performance was further improved compared to the model that uses the WMF method with multi-step training. This showed that WMF fully utilizes global information and local details by weighted fusion of features from multiple layers, further enhancing the model’s classification ability and improving its adaptability to complex samples. Additionally, in this evaluation, “p1”, “p2”, and “p3” refer to the classification accuracy at different stages of the ResNet-50 backbone, corresponding to the third, fourth, and fifth convolutional blocks, respectively. These intermediate predictions offer insight into the feature representation quality at various network depths.

**Figure 4:**
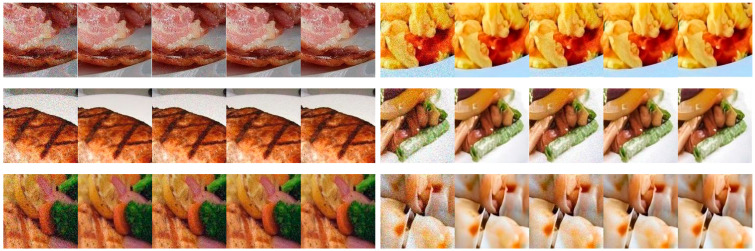
Visual analysis of noise reduction effect. The three sets of images on the left are from the ETH Food-101 dataset, and those on the right are from the Vireo Food-172 dataset. In each collection of five images, the progression from left to right is shown: starting with the image that has added Gaussian noise, followed by the original image, and concluding with the results obtained after three rounds of NARM denoising. All images are uniformly cropped to 150 × 150 size for display.

**Figure 5:**
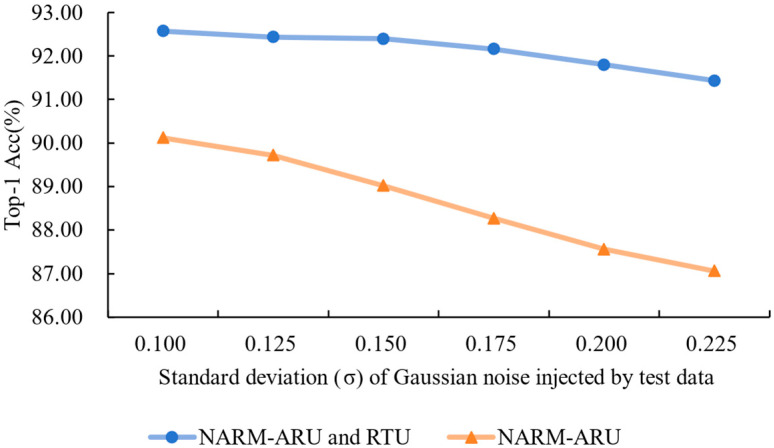
The accuracy performance of the NARM model with RTU and the NARM model with ARU is compared when different intensity noise is injected into the test data.

RTU Noise Invariance Validation Experiment: To verify the effectiveness of the proposed Residual Transformation Unit (RTU) in achieving noise invariance, we initially evaluate the performance of the three integrated NARMs for image denoising tasks within the backbone network. As shown in Figure 4, processing through the three NARMs generates a relatively smooth denoised image for a noisy image. This result indicates that our model can effectively capture noise invariance. Furthermore, to further validate RTU’s advantages, we compared the NARM model integrated with RTU (NARM-ARU and RTU) with the control model that contains only ARU (NARM-ARU). For the ETH Food-101 dataset, we injected different noise levels into the test data and recorded the classification accuracy of the two models under different noise intensities. The results are shown in Figure 5. As the noise intensity increases, the model with RTU experiences a slower decline in accuracy, while the model without RTU shows a sharp decrease in accuracy. These experimental results effectively demonstrate the significant advantages of RTU in noise handling.

**Figure 6:**
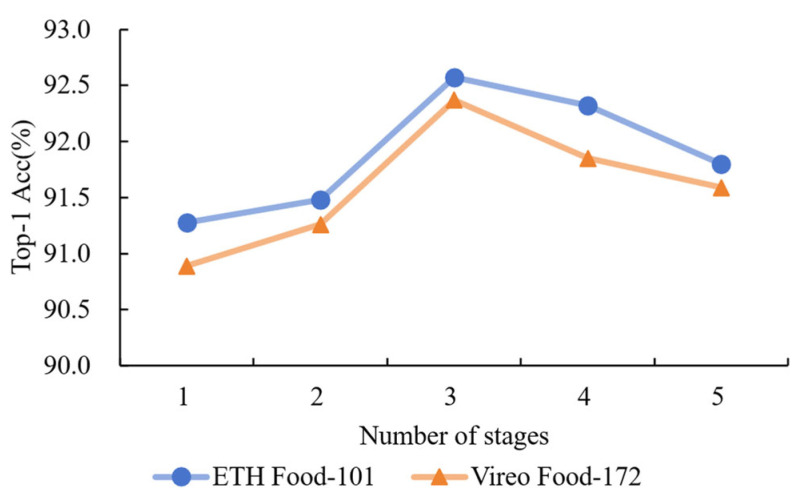
Comparison of the effects of different stages in backbone network selection reveals that features from the final three stages yield the best results.

Network Layer Design Optimization Experiment: To evaluate the model’s effectiveness, we compared the impact of inserting NARMs at different stages on performance. The experiment used ResNet50 as the base network, with NARMs inserted at the last one, two, three, four, and all five stages. The experimental outcomes, as illustrated in Figure 6, demonstrate that the model incorporating NARMs in the final three stages attained superior performance on both the ETH Food-101 and Vireo Food-172 datasets. In comparison, the model with NARMs inserted only at the last stage exhibited lower accuracy due to the absence of multi-level feature support. Although inserting NARMs in the previous two stages showed some improvement, it failed to utilize features from additional layers fully. When inserted up to the last four stages, the accuracy improved to 92.32% and 91.85% on the Food-101 and Vireo Food-172 datasets, respectively, but the gain leveled off. Lastly, when NARMs were inserted at all five stages, classification performance was slightly improved, but the training time and computational resource consumption increased. In summary, inserting NARMs in the last three stages provides the best balance between performance and computational complexity, effectively utilizing multi-level features.

Computational Efficiency Evaluation Experiment: To assess the computational overhead introduced by the proposed modules, we conducted comparative experiments on the ETH Food-101 dataset using the SFF baseline and models integrated with NARM, WMF, and PTAFD. All models were trained under consistent conditions as described earlier. As summarized in Table 3, the parameter count increased from 25.5 million in SFF to 33.2 million after integrating NARM, primarily due to the dual-task design. Adding WMF further raised the parameter count to 41.5 million, while the PTAFD student model remained compact at 25.7 million. In terms of training time, SFF required 8.1 min per epoch, which increased to 10.6 min with NARM and 14.0 min with WMF. PTAFD remained efficient at 8.4 min per epoch. Peak memory usage also rose from 14.5 GB in SFF to 20.8 GB in the full model, while PTAFD required only 15.0 GB. Overall, the teacher model with NARM and WMF achieved the highest accuracy on ETH Food-101 but at the cost of increased computational demand. In contrast, the PTAFD student model maintained strong performance while offering greater training efficiency and hardware compatibility.

**Figure 7:**
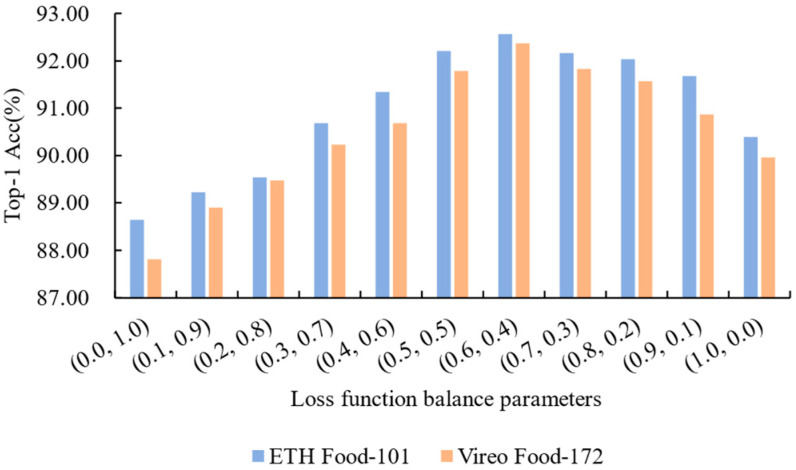
The analysis of how different balancing parameters affect experimental outcomes reveals that relying solely on either loss function $L_{\mathrm{rec}}$ or $L_{\mathrm{mse}}$ results in suboptimal performance. The highest recognition accuracy is attained when the balancing parameters are configured to (0.6, 0.4).

Loss Function Parameter Sensitivity Experiment: The model proposed in this paper uses a weighted loss function composed of classification loss $L_{\mathrm{rec}}$ and recovery loss $L_{\mathrm{mse}}$ in the backward propagation of the last three layers to optimize classification performance and denoising recovery ability. The experiment tested the impact of different weight settings on model performance. The results, shown in Figure 7, indicate that when (α, β) = (0.6, 0.4), the model performed best on both datasets, validating the necessity of appropriate weight allocation between classification and recovery performance. Specifically, the weight settings reflect the dominant role of the recognition task, with classification being the core function and requiring a higher weight (α>β), while denoising, as a secondary task, holds relatively less importance. Further analysis revealed that when β = 0 (only optimizing classification loss), the model neglects noise recovery, resulting in uncorrected biases that affect classification performance. When α = 0 (only optimizing recovery loss), while low-level visual features are well restored, the lack of high-level semantic enhancement limits the classifier’s effectiveness. This indicates that classification loss and recovery loss optimize the model from high-level semantics and low-level recovery perspectives, complementing each other. Optimizing a single loss leads to biased feature representation, thus affecting overall performance. Through weighted combination, the model achieves a balance between classification performance and noise robustness, significantly improving performance in complex noise scenarios.

Per-Class Performance Analysis of Ablation Modules: To provide a fine-grained performance analysis beyond overall metrics, we conducted ablation experiments on the full ETH Food-101 and Vireo Food-172 datasets, comparing the complete model with variants lacking EGCP, WMF, or both. This detailed evaluation, placed after the global performance and parameter analysis, further reveals how each module affects recognition across diverse food categories, including visually similar, noise-sensitive, and minority classes. Table 4 presents results from the first ten categories in ETH Food-101 to illustrate the observed effects. The full model achieves an average F1-score of 91.2% and recall of 90.8%. Removing EGCP causes an average drop of 3.2% in F1 and 3.5% in recall, with particularly notable declines in beef carpaccio and beef tartare, where fine-grained texture distinctions are crucial. This confirmed EGCP’s role in enhancing subtle structural cues under noise interference. Excluding WMF leads to reduced performance in visually diverse dishes such as bibimbap and breakfast burrito, where multi-scale fusion is essential for capturing complex visual patterns. When both modules are removed, performance drops more significantly, especially in visually rich categories like baklava and beignets. These results highlight the complementary strengths of EGCP and WMF and provide detailed insight into model behavior across challenging food classes.

### 3.3. Effect of Noise Intensity

To evaluate the model’s robustness under varying noise conditions, we performed a controlled study on the effect of Gaussian noise intensity. Specifically, we added additive zero-mean Gaussian noise to the training images, where the noise was applied pixel-wise to each RGB channel independently. The noise followed a normal distribution $N(0,\sigma^{2})$, where σ denotes the standard deviation controlling the noise intensity.

The noise injection was performed only during the training phase, while all test images remained clean to simulate real-world scenarios where models must generalize from noisy training data to clean inputs. We experimented with five noise intensity levels by setting σ to 0.01, 0.05, 0.10, 0.15, and 0.20, respectively. For each setting, the entire training set was augmented with noise at the specified level.

This process simulates sensor or lighting-related degradation commonly encountered in food photography, such as low-light blur or camera grain. The model was retrained from scratch under each noise condition using the same initialization, optimization schedule, and data splits to ensure fair comparison. All noise samples were generated using a fixed random seed for reproducibility.

**Figure 8:**
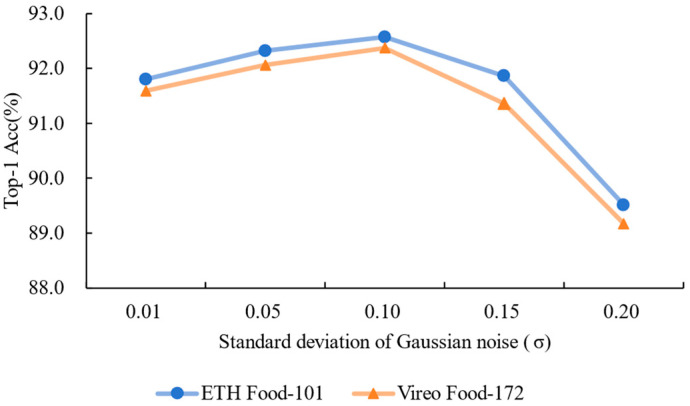
Impact of Gaussian noise intensity on classification accuracy for different datasets (Highest accuracy achieved at σ=0.10. This can be attributed to the regularization effect of moderate noise injection, which encourages the model to learn more generalizable, noise-invariant features. However, beyond σ=0.15, the distortion outweighs the benefit, leading to degraded performance.).

Experiment on the Effect of Different Gaussian Noise Intensities: Gaussian noise is injected into the original images as input in the proposed method. In this experiment, Gaussian noise with different standard deviations (σ) is injected into the NARM, with specific values of [0.01, 0.05, 0.10, 0.15, 0.20]. By comparing the model’s classification accuracy under different noise levels, we analyze the effect of noise intensity on noise-robust learning and feature enhancement. The experimental results are shown in Figure 8. As the noise intensity increases, the model’s classification accuracy first rises and then declines. When the noise intensity is σ=0.10, the model exhibits the best noise robustness and classification performance, with a significant increase in accuracy. However, when the noise intensity further increases (σ>0.15), the interference from noise on the model’s feature representation becomes dominant, leading to a significant drop in classification performance. Considering both noise-robust performance and classification accuracy, we ultimately choose σ=0.10 as the standard deviation for injecting Gaussian noise. We will use this parameter in subsequent experiments for further model optimization and validation.

### 3.4. Evaluation of Distillation Strategies

The PTAFD method is designed as a lightweight yet effective knowledge distillation (KD) strategy. This experiment demonstrated its superiority as a teacher network compared to other advanced KD methods, including Relational Knowledge Distillation (RKD) [48], Correlation Congruence (CC) [49], Instance Relation Graph (IRG) [50], Attention-Based Distillation (AB) [51], Learning without Memorizing (LWM) [52], Feature-Based Progressive Distillation (FPD) [53], and Two-Stage Knowledge Distillation (TSKD) [54]. We used a high-capacity teacher model and a ResNet50-based student model to assess the impact of different KD techniques on student model performance.

KD methods can generally be divided into two categories: the first focuses on aligning point-to-point features of the student model with those of the teacher, typically by guiding the student to learn intermediate representations or selectively distill informative features to enhance training (e.g., FPD, AB, IRG, and LWM), and PTAFD also belongs to this category. The second category aims to align feature distributions or relational structures between teacher and student models, often through distance-based or angular loss minimization (e.g., RKD, TSKD, and CC).

**Figure 9:**
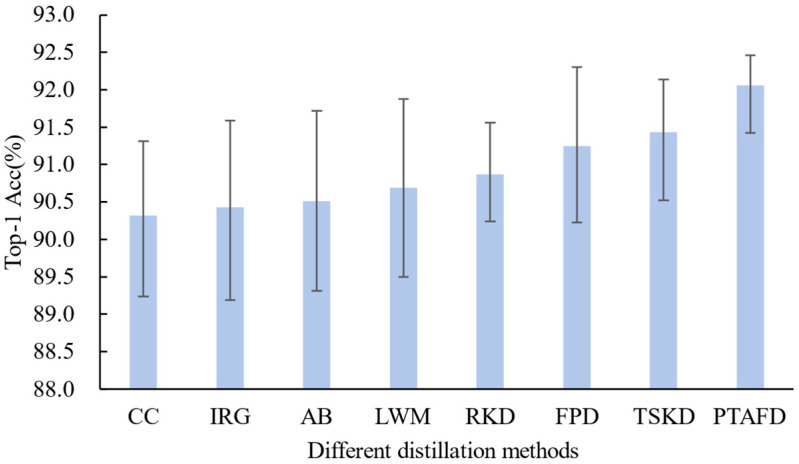
Comparison of distillation performance of different distillation methods and PTAFD on the ETH Food-101 dataset. Error bars indicate the standard error of the mean (SEM) computed from five runs, and the top of the upper error line and the bottom of the lower error line represent each method’s maximum and minimum values, respectively. The comparison includes feature alignment-based methods, such as CC [49], IRG [50], AB [51], LWM [52], and FPD [53], and feature distribution alignment methods like RKD [48], TSKD [54], and PTAFD.

Figure 9 compares the distillation performance of these KD methods with PTAFD on the ETH Food-101 dataset. All models were trained five times independently using each method, and the results are presented using error bar plots as follows: the central bar indicates mean accuracy, while the upper and lower bars represent the maximum and minimum values, respectively. On average, the student model trained with PTAFD achieved 0.63–1.74% higher Top-1 accuracy than those trained with other KD methods, indicating more effective knowledge transfer.

## 4. Discussion

This study proposed a novel food image recognition framework that integrates anti-noise learning and covariance feature enhancement. The experimental results demonstrate its state-of-the-art performance on multiple datasets. The effectiveness of the proposed architecture stems from the synergy among its key modules as follows: the NARM enhances noise resilience through dual-task learning; EGCP improves fine-grained recognition by amplifying small eigenvalue information; WMF enables adaptive multi-level feature integration; and PTAFD facilitates knowledge transfer for efficient deployment. These modules collectively enhanced robustness against noise and variability in food categories, enabling accurate recognition even under real-world conditions.

To further contextualize our results, we compared our model with recent benchmarks summarized by Liu et al. [55], whose supplementary Table S5 reported the performance of several deep learning-based food recognition models. For example, CBiAFormer and SLCI achieved a Top-1 accuracy of 92.40% and Top-5 accuracy of 98.87% on ETH Food-101, as well as 91.58% (Top-1) and 98.75% (Top-5) on Vireo Food-172. In comparison, our proposed method achieved slightly better results, with a Top-1 accuracy of 92.57% and Top-5 accuracy of 99.03% on ETH Food-101, and 91.72% (Top-1) and 98.81% (Top-5) on Vireo Food-172. These outcomes confirm that our model not only attains state-of-the-art classification accuracy, but it also exhibits greater robustness—particularly under noisy and fine-grained food categories. Unlike many existing methods such as CBiAFormer, which primarily focus on clean datasets, our approach maintains consistent performance on real-world data, as further demonstrated in Section 3.3. This highlights the practical value and broad applicability of the proposed framework in real-world food recognition scenarios.

Despite its strong performance, several limitations exist. First, the RTU component shows performance degradation under severe noise levels (σ>0.2), indicating that future improvements may require more advanced denoising techniques. Second, the generalizability of the model needs further validation on more diverse datasets, including those with extreme lighting, occlusions, or underrepresented food types. Third, although PTAFD improves efficiency, WMF introduces computational overhead, which could limit applicability on resource-constrained devices.

Future work will focus on addressing these limitations by incorporating transformer-based restoration models, expanding benchmark datasets, and developing lightweight variants suitable for mobile deployment. These enhancements aim to further improve the model’s applicability in real-world food computing scenarios.

## 5. Conclusions

We proposed a robust food image recognition framework that effectively addresses noise interference and fine-grained classification challenges. By combining the NARM, EGCP, WMF, and PTAFD modules, the model achieves state-of-the-art Top-1 accuracy (92.57%) on ETH Food-101 while maintaining high efficiency and generalization across datasets. In particular, the integration of NARM, EGCP, and WMF with the ResNet-50 backbone demonstrates the best robustness under Gaussian noise perturbations, confirming its effectiveness in noisy real-world environments. These findings highlighted the method’s practical potential in dietary assessment and intelligent food systems.

However, this study has some limitations. The model’s robustness under complex real-world conditions such as varying lighting and occlusions in mobile-captured images remains to be further validated. In future work, we plan to evaluate performance on user-generated food datasets and explore strategies to improve generalization to unseen food categories.

## Figures and Tables

**Table 1:** Comparison of various food recognition methods on the ETH Food-101 and VIREO Food-172 datasets (%).

Method	Backbone	ETH Food-101		Vireo Food-172	
		Top-1 Acc	Top-5 Acc	Top-1 Acc	Top-5 Acc
ResNet152 + SVM-RBF [38]	ResNet152	64.98	-	-	-
FS_UAMS [39]	Inceptionv3	-	-	89.26	-
ResNet50 [16]	ResNet50	87.42	97.40	-	-
DenseNet161 [33]	DenseNet161	-	-	86.98	97.31
SENet-154 [40]	ResNeXt-50	88.68	97.62	88.78	97.76
PAR-Net [41]	ResNet101	89.30	-	89.60	-
DCL [42]	ResNet50	88.90	97.82	-	-
PMG [43]	ResNet50	86.93	97.21	-	-
WS-DAN [44]	Inceptionv3	88.90	98.11	-	-
NTS-NET [45]	ResNet50	89.40	97.80	-	-
PRENet [34]	ResNet50	89.91	98.04	-	-
PRENet [34]	SENet154	90.74	98.48	-	-
SGLANet [46]	SENet154	89.69	98.01	90.30	98.03
Swin-B [28]	Transformer	89.78	97.98	89.15	98.02
DAT [47]	Transformer	90.04	98.12	89.25	98.12
EHFR-Net [20]	Transformer	90.70	-	90.30	-
IVRDRM [35]	ResNet-101	92.36	98.68	**93.33**	**99.15**
SICL(CBiAFormer-T) [29]	Swin-T	91.11	98.63	90.70	98.05
SICL(CBiAFormer-B) [29]	Swin-B	92.40	**98.87**	91.58	98.75
Our method *	ResNet50	**92.57**	98.70	92.37	98.55

* Our method refers to the proposed model that integrates SFF, NARM, and WMF modules, consistent with “SFF + NARM + WMF” in Table 2. (Notes: Values in bold indicate the highest accuracy in each respective column. Bracketed numbers refer to reference entries in the bibliography corresponding to the cited algorithms or models).

**Table 2:** Ablation study on the contributions of NARM and WMF (Top-1 Accuracy, %).

	ETH Food-101				Vireo Food-172			
	p_1_	p_2_	p_3_	Top-1	p_1_	p_2_	p_3_	Top-1
SFF	86.43	87.23	86.79	87.86	82.87	86.12	85.72	86.63
SFF + NARM (no EGCP)	87.73	88.36	88.97	90.31	86.58	87.65	88.67	89.72
SFF + NARM	89.25	90.80	91.23	92.19	88.23	89.82	91.10	91.88
SFF + NARM + WMF	89.77	91.27	92.03	**92.57**	88.69	90.03	91.59	**92.37**

**Table 3:** Comparative analysis of computational costs before and after module integration.

Model	Param (M)	Epoch Time (min)	Total Time (h)	Memory (GB/GPU)	Top-1 (%)
SFF	25.50	8.10	27.00	14.50	87.42
SFF + NARM	33.20	10.60	35.30	17.30	92.19
SFF + NARM + WMF	41.50	14.00	46.70	20.80	**92.57**
SFF (PTAFD Student Model)	25.70	8.40	28.00	15.00	92.01

**Table 4:** Per-class performance analysis of model variants in ablation experiments.

Class	Our Method		Our Method w/o EGCP		Our Method w/o WMF		Our Method w/o EGCP and WMF	
	Recall	F1-Score	Recall	F1-Score	Recall	F1-Score	Recall	F1-Score
Apple pie	92.37	91.84	88.72	88.25	89.56	89.03	86.19	85.62
Baby back ribs	93.12	92.76	89.84	89.21	90.23	89.77	87.05	86.58
Baklava	91.08	91.53	87.31	87.06	88.52	88.39	83.87	84.29
Beef carpaccio	89.53	89.07	84.15	83.92	86.38	86.09	81.24	81.06
Beef tartare	88.91	88.65	83.97	83.84	85.72	85.43	80.59	80.32
Beet salad	90.76	90.28	87.36	86.89	88.15	87.64	84.63	84.17
Beignets	92.58	92.14	88.92	88.57	89.86	89.45	85.92	85.23
Bibimbap	90.25	89.83	86.04	85.58	85.79	85.62	82.37	81.95
Bread pudding	91.84	91.36	88.17	87.75	88.93	88.56	85.08	84.62
Breakfast burrito	91.27	90.85	87.06	86.53	86.45	86.07	83.15	82.74
Average	90.82	91.21	87.33	88.01	87.91	88.51	84.01	84.63

## Data Availability

The experimental data used in this study are publicly available at the ETH Food-101 (https://data.vision.ee.ethz.ch/cvl/datasets_extra/food-101/, accessed on 1 July 2025) and Vireo Food-172 (https://fvl.fudan.edu.cn/dataset/vireofood172/list.htm, accessed on 1 July 2025) datasets. The source code is not publicly released due to institutional restrictions. However, interested researchers may contact the corresponding author to request access. Upon reasonable evaluation, relevant components of the code may be provided to support reproducibility.
